# Feasibility and accuracy of targeted axillary dissection in breast cancer patients; single center experience

**DOI:** 10.3389/fsurg.2023.1332142

**Published:** 2024-01-10

**Authors:** Cemal Kaya, Büşra Burcu, Işık Çetinoğlu, Ramazan Uçak, Esma Çerekçi, Cennet Şahin, Zeynep Gül Demircioğlu, Aziz Şener, Süleyman Halil

**Affiliations:** ^1^ Department of General Surgery, Şişli Hamidiye Etfal Education and Research Hospital, İstanbul, Türkiye; ^2^ Department of Pathology, Şişli Hamidiye Etfal Education and Research Hospital, İstanbul, Türkiye; ^3^ Department of Radiology, Şişli Hamidiye Etfal Education and Research Hospital, İstanbul, Türkiye; ^4^ Department of Medical Oncology, Şişli Hamidiye Etfal Education and Research Hospital, İstanbul, Türkiye

**Keywords:** clip-on lymph node, neoadjuvant chemotherapy, targeted axillary dissection, breast cancer, sentinel lymph node

## Abstract

**Introduction:**

Axillary complete response (pCR) was observed in approximately half of breast cancer patients who received neoadjuvant chemotherapy (NAC) due to axillary positivity. Preventing axillary morbidity due to unnecessary axillary lymph node dissection (ALND) is extremely important for patients' quality of life. Targeted axillary dissection (TAD) is a technique developed to improve axillary staging and reduce the false negative rate in sentinel lymph node biopsy.

**Methods:**

Patients with cN1-N2 (clinically node) breast cancer whose axillary lymph node metastasis was confirmed by biopsy and who received NAC marked with a clip were included in the study. Patients who achieved clinical response after systemic treatment underwent sentinel lymph node biopsy (SLNB) with additional methods such as methylene blue guided dissection, skin marking for clip on lymph node (CLN) localization, and wire guided with imaging excision of the CLN. TAD and ALND pathology results were evaluated and analyzed with patient and tumor characteristics.

**Results:**

83 patients who met the eligibility criterias for the study were examined retrospectively. 21 of the patients underwent TAD alone, and 62 patients underwent ALND after TAD. CLN rate was 98.8% in patients underwent only TAD and this rate was increased to 100% in patients underwent ALND after TAD. FNR in SLN, CLN, and TAD were 28.6%, 10.7%, and 3.6%, respectively.

**Conclusion:**

TAD is a feasible and reliable surgical approach to detect axillary residual disease with a high success rate.

## Introduction

Nowadays, neoadjuvant chemotherapy (NAC) is frequently used for locally advanced tumors, most triple-negative and HER2+ tumors, and a significant portion of HR+HER2- lymph node-positive tumors. Indications of NAC include tumor downsizing to facilitate breast-conserving surgery (BCS) and also nodal stage regression (1). Providing less radical surgery, evaluation of treatment effectiveness, and prognosis prediction are the most important advantages of NAC.It may be possible to avoid ALND in patients with positive lymph nodes by ensuring clinical negativity after systemic treatment. Studies have shown that the use of SLNB after NAC allows avoiding ALND in more than 40% of cases (2).

The reason for the high FNR after NAC was thought to be fibrosis in the lymphatics following chemotherapy, treatment of tumor embolism, which causes lymphatic obstruction, and blockage of the lymphatics to which the tumor develops (3). It could causes to 8%–40% of positive lymph nodes being missed in patients despite negative SLN (4).

The ACOSOG (American College of Surgeons Oncology Group) Z1071 and SENTINA (SENTinel NeoAdjuvant) studies have shown that FNR can be reduced in this group of patients if two or more lymph nodes are removed and a dual-agent mapping technique is used (5, 6). Further studies on this subject have shown that this rate may decrease below 10% in patients for whom NAC is planned, when positive lymph nodes are clipped before treatment and removed at the end of treatment (7, 8).

The TAD procedure is the removal of SLN and CLN after NAC and has been widely performed in recent years with FNR rates as low as 2% (8–10).

Our aim in this study is to investigate the applicability of the accuracy and reliability of TAD, which has been widely used in recent years, in axillary staging in the clinical practise.

## Material and methods

This is a single-center, retrospective, observational study. Patients who received treatment for breast cancer diagnosis at the General Surgery Clinic of Şişli Hamidiye Etfal Training and Research Hospital between July 2016 and February 2023 and received NAC by the decision of the breast council were included in study. After ethics committee approval (2273/30.05.23) was obtained, patients' surgical, clinical, radiology, and pathology reports were collected.

### Inclusion—exclusion criterias

The patients with T1-3 grade tumor and axillary N1-2 lymph nodes metastasis marked with metallic clip before NAC and had achieved cN0 on physical examination and radiologic controls after systemic therapy were included in the study. Patients with N3 nodal metastases, patients who did not achieve cN0 after systemic therapy, patients with recurrence or distant metastasis, patients with inflammatory and bilateral breast cancer, patients who underwent axillary surgery (e.g., SLNB or nodal sampling) before NAC and pregnant breast cancer patients were excluded.

### Marking lymph nodes

**Figure 1:**
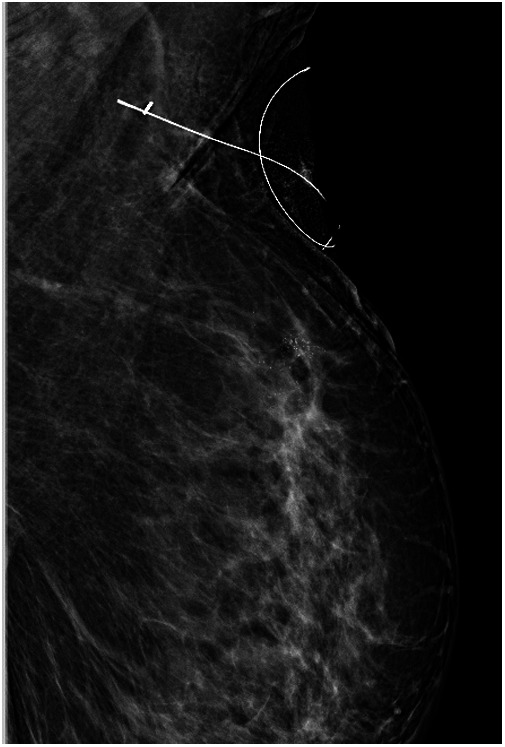
The mammography imaging confirming the metallic clip localization under the wire guidance.

**Figure 2:**
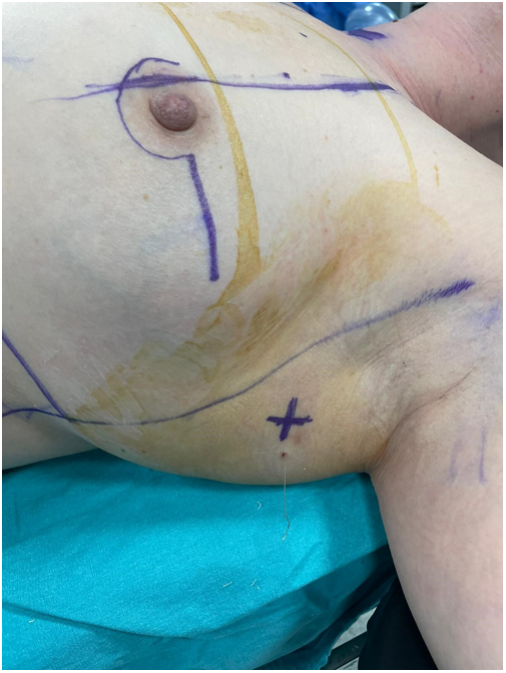
The skin projection (X symbol) of the lymph node localized with wire.

All radiologic procedures were performed by two radiologists experienced in breast radiology and interventional procedures. The cortex of the biopsy-confirmed axillary metastatic lymph node was marked with a metallic clip (Geotek, Turkey®) with ultrasonography (USG) guidance (Mindray, China; Siemens Healthcare, Erlangen, Germany) with a 5.5–18 MHz linear probe before NAC. The clip was also placed in the 2nd most suspicious lymph node from the N2 group lymph nodes. After the procedure, the metallic clip localization was confirmed by mamography (MM) (Figure 1). The marker in these lymph nodes was checked by USG, MM and/or computed tomography (CT) imaging after NAC in the week before surgery. On the day of surgery, the clipped lymph nodes were stereotactically wired to localize them with USG or CT guidance. At the same time, the skin projection of the clip localization was marked as X symbol with a blue pen, mimicking the surgical position (Figure 2).

### Surgical procedure

During the surgery, SLNB was performed using the periareolar subcutaneous injection technique with 5 cc methylene blue (Blumet, Vem İlaç®). At the same time, the CLN marked with a wire was excised by stereotactic biopsy through an incision made from the skin projection mark on it. All lymph nodes stained with methylene blue were accepted as SLN. After the mammographic images of all removed CLNs, specimens were sent for pathological examination together with the stained lymph node(s). Mastectomy or BCS techniques were performed for surgical treatment, according to the tumor and the patient's characteristics and preferences.

### Pathological examination

Lymph nodes stained with methylene blue were considered SLN, and lymph nodes marked with marker-wire were considered CLN. In the peroperative frozen examination, if the lymph node size was less than 0.5 cm, it was evaluated in one piece; if it was between 0.5 cm and 1 cm, it was evaluated in two slices. If it was larger than 1 cm, it was divided longitudinally at 2 mm intervals and assessed by touch imprint or squash preparations. Some rare cases were examined by postoperative frozen section due to the inability to reach a decision. Metastatic lymph nodes were evaluated according to the College of American Pathologists (CAP) protocol, and definitive data were reported by microscopic examination of lymph node sections. Tumor infiltration greater than 2 mm was accepted as macrometastases; tumor infiltration of 0.2 mm to 2 mm and/or 200 cells was accepted micrometastases; tumor infiltration of ≤0.2 mm and ≤200 cells was accepted isolated tumor cells.

### Statistical method

SPSS 15.0 for Windows was used for statistical analysis. As descriptive statistics, number and percentage were given for categorical variables, and mean, standard deviation, minimum, and maximum were given for numerical variables. A comparison of rates in dependent groups was made with the McNemar test. The agreement between the evaluations was given by the Kappa coefficient. The statistical alpha significance level was accepted as *p* < 0.05.

## Results

The data of 145 patients who underwent axillary node marking with clip before NAC were analyzed. 11 patients with insufficient follow-up data and 32 patients who underwent direct ALND because cN0 could not be obtained at the end of systemic treatment were excluded from the study. 19 patients did not have lymph node staining in the SLNB procedure with methylene blue underwent direct ALND because of failed axillary mapping and excluded. A total of 83 patients were included in the study and their clinicopathologic characteristics were shown in Table 1. Only TAD was performed to 21 patients, and ALND was performed together with TAD to 62 patients.

**Table 1:** The clinicopathological features of the patients.

Age mean ± SD (min–max)	50.5 ± 9.2 (32–78)
Histology *n* (%)	
Ductal	68 (81.9)
Lobuler	8 (9.6)
Other	7 (8.4)
Grade *n* (%)	
1	14 (16.9)
2	47 (56.6)
3	22 (26.5)
Lymphovascular invasion (LVI) *n* (%)	
Yes	47 (56.6)
No	36 (43.4)
Molecular type *n* (%)	
Luminal A	54 (65.1)
Luminal B	10 (12.0)
HR+/HER2+	7 (8.4)
HR-/HER2+	7 (8.4)
TN	5 (6.0)
N stage *n* (%)	
N1	61 (73.5)
N2	22 (26.5)
T stage *n* (%)	
T1	17 (20.5)
T2	63 (75.9)
T3	3 (3.6)
Number of clipped lymph nodes *n* (%)	
1	71 (85.5)
2	12 (14.5)
Surgery *n* (%)	
Mastectomy	37 (44.6)
BCS	46 (55.4)

The detection rate of SLN was 81.4% (83/102) and the mean of SLN was 2.5 (1–5). The rate of negative and positive SLN were 53% (*n* = 44) and 47% (*n* = 39), respectively (Table 2). The difference in the rates of negative and positive SLN was statistically significant (*p* = 0.001). Statistical correlation between the results was moderate (Kappa coefficient 0.480). In patients with SLN and CLN in the same lymph node, the difference in negative-positive rates was statistically significant and the correlation was poor (*p* = 0001, Kappa coefficient 0.394). No statistically significant difference was detected in patients with different SLN and CLN lymph nodes, and the correlation was moderate (Kappa coefficient 0.594) (Table 3).

**Table 2:** The negativity and positivity rates of the sentinel and clip on lymph nodes.

	SLN negative	SLN positive	Total
CLN negative	25 (30.1%)	3 (3.6%)	28 (33.7%)
CLN positive	19 (22.9%)	36 (43.4%)	55 (66.3%)
Total	44 (53.0%)	39 (47.0%)	83

**Table 3:** Statistical concordance between the sentinel and clip on lymph nodes.

	Clip-Dyed LN		
	Same *n* = 47	Different *n* = 36	Total
CLN&SLN concordance	32 (68.1%)	29 (80.5%)	61 (73.5%)
McNemar test *p*	0.007	0.125	0.001
Measure of agreement Kappa	0.394	0.594	0.480

Single clip was placed in 71 patients and double clips were placed in 12 patients. In 10 patients with double clips, both clips were removed with TAD, and in 2 patients, the second clip was removed with ALND. Although the clip was removed with stereotactically in 70 patients with a single clip, in one patient clip could have not removed with stereotactically and ALND was performed for removing clip. In the preoperative evaluation, the clip was localized in all patients, and the detection rate was found to be 98.6% for a single clip in TAD, 100% for one clip in patients with two clips, and 97.6% for the second clip. When patients with double clips were evaluated among themselves, false negativity was detected in the SLN in one patient, while no false negativity was detected in the CLN and TAD.

Of the 62 patients who underwent ALND, 28 (33.7%) had positive axilla and 3 had negative axilla. In the other 31 patients who underwent ALND, no positive lymph node was found in the axilla except for SLN and/or CLN. Axillary pCR rate was 28.9% (*n* = 21 + 3). A total of 83 patients underwent TAD procedure and 62 of them also underwent ALND. When the positive lymph nodes (*n* = 28) in 62 patients who underwent ALND after TAD were analyzed, it was seen that 3 patients had CLN, 8 patients had SLN and 1 patient had negative TAD (SLN + CLN). False negativity rates according to positive axillary dissection results were 28.6%, 10.7% and 3.6% for SLN, CLN and TAD, respectively (Table 4).

**Table 4:** False negative rate of CLN, SLN and TAD.

	Positive axillary dissection *n* = 28 (33.7%)	McNemar test *p*	Measure of agreement Kappa
CLN neg.	3 (10.7%)	<0.001	0.281
SLN neg.	8 (28.6%)	0.052	0.336
TAD (SLN + CLN) neg.	1 (3.6%)	<0.001	0.317

Translated with DeepL.com (free version) False negativity rates according to positive axillary dissection results were 28.6%, 10.7% and 3.6% for SLN, CLN and TAD, respectively (Table 4).

45.8% (*n* = 38) of the patients underwent BCS and 54.2% (*n* = 45) underwent mastectomy (subcutaneous mastectomy with implant reconstruction 11 patients). In our study, except for simple hematoma in 3 patients at the clip placement stage, no other clip or wire-related complications were reported.

## Discussion

The pCR provided by NAC in axilla-positive breast cancers is important for prognosis prediction as well as for axilla-preserving surgery. Less invasive staging methods have been investigated to identify these patients and avoid unnecessary ALND. For this purpose, methods such as SLNB, MARI procedure (marking the axillary lymph node with radioactive iodine seed) and TAD have been applied (5, 6, 9, 11).

However, in these patients, there is a delicate balance between avoiding dissection and ensuring oncological safety in order to reduce axillary morbidity. The ACOSOG Z1071 study reported that FNR was 12.6% in the case of SLNB alone in this patient group (6). Since the impact of FNR above 10% on clinical and oncological outcomes in these patients remains unclear, some national guidelines continued to recommend ALND in these patients [(12–14) 18, 19, 20]. Moreover, in the subanalysis of the ACOSOG Z1071 study and the SENTINA study, it was determined that the FNR rate decreased below 10% if the dual method was used and more lymph nodes were removed (5, 6).

The European Society for Medical Oncology (ESMO) and the National Comprehensive Cancer Network (NCCN) state that ALND may not be performed if positive nodes are marked and more than 2 SLNs are removed (15, 16). In the TAD procedure, the marking and localization of pathological LAPs in the axilla could appiled with various techniques. For this purpose, marking and localization techniques such as wire, skin projection, I125-labeled radioactive seed, and intraoperative USG could be used after placing the clip (9, 17–20).

On the other hand, there is no consensus for choosing the most optimal of these techniques. All these techniques have various advantages and disadvantages, such as institution resources, cost effectiveness, radiation exposure, compatibility with surgical instruments, differences in identification rates (IR). In the literature, the most commonly used method is the clip marking and guide wire localization technique, with a clip-on lymph node IR of over 95% (21–23).

In breast cancer patients who are planning to undergo NAC, we mark and localize the metastatic lymph node with a clip using the clip and wire localization technique in accordance with the conditions of our institution. In our clinic, clips are routinely placed on patients with N1 and on some of patients with N2 metastatic lymph nodes before NAC. At the end of systemic treatment, they are checked with USG and MM one week before the surgical operation. The clip is localized with a wire and skin markings placed with the appropriate imaging method one hour before the operation. We also use MM or CT to confirm the clip in the lymph node. In the literature, it has been stated that CT is also used for wire localization (24, 25).

In our study, we placed one clip in N1 patients, double clips in N2 patients for the most suspicious node other than the one identified by biopsy. Although there is lack of data in the literature about the number of clips to be placed, placing clip to the positive LN provides better oncological results despite the cost and procedure disadvantages due to tumor heterogeneity in our opinion.

**Figure 3:**
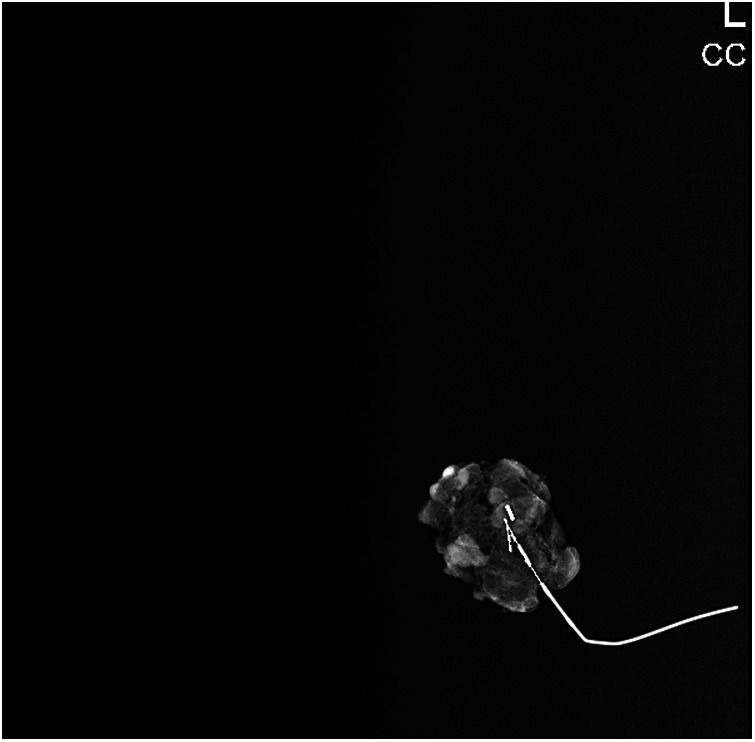
The removed clip on lymph node confirmed via mammography imaging.

Among our patients, the number of patients who underwent TAD alone was 21 (25.3%), and the number of patients who underwent ALND after TAD was 62 (74.7%). The IR of the clipped lymph node and removal of the clip was quite high and was found to be 98.6% for a single clip in stereotactically and 100% with ALND in our study. In our clinic, specimen MM is routinely taken to check whether there is a clip in the lymph node removed with wire localization. In cases where there is no staining and the clip cannot be found, ALND is performed and the presence of the clip in the sample is re-checked radiologically (Figure 3). We believe that removing the clips completely is an essential issue, as the clips left behind may create legal as well as medical problems. In our hospital, we work in coordination with radiology during the clip placement, localization, and removal phases. Performing the biopsy and clipping by the same radiologist increases the success of the procedure. We routinely mark the skin projection along with wire placement during the clip localization phase. In a study about clip localization, the detection rate of LN was reported as 100% when wire and skin markings were used (20).

In our study, the detection rate of SLN with methylene blue alone was calculated based on 102 patients, this rate was found to be 81.4%. In literature, many various values were given regarding the rate of SLN in patients recieved NAC. In a meta-analysis, IR was calculated in the range of 86%–100% in patients who underwent SLNB using methylene blue after NAC (26). We think that the skin marking technique together with the wire placement for clip localization in the patients in our study contributed to the detection rates of SLNB.

**Figure 4:**
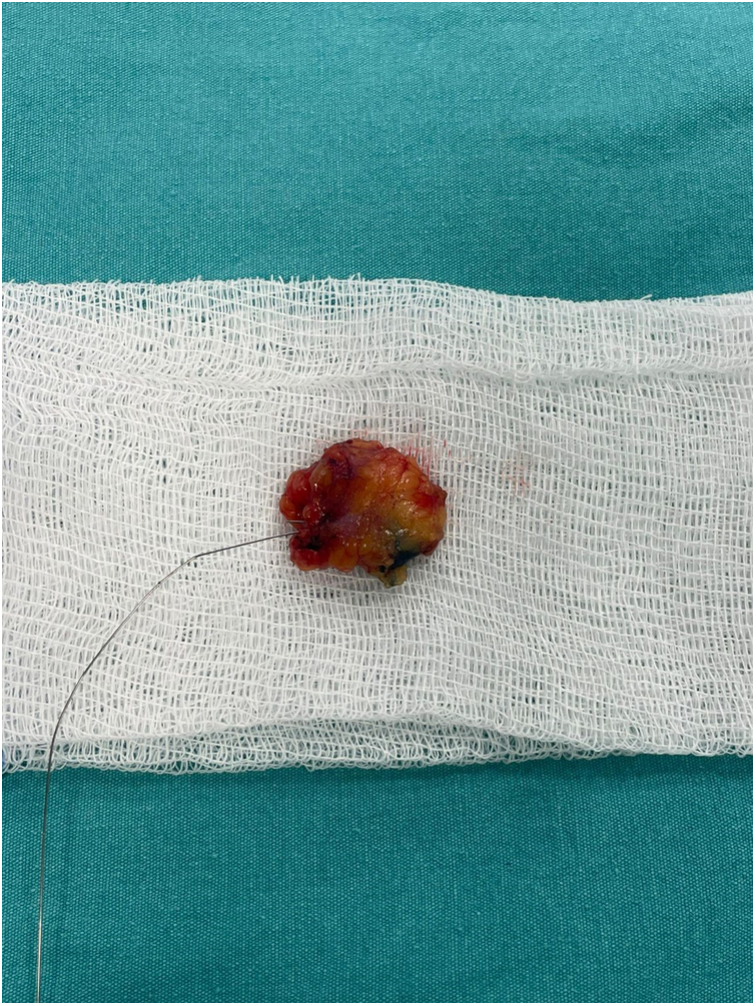
The removed lymph node demonstrating both the stain and the clip.

Regarding the rates given in the literature on whether SLN and CLN are similar, this rate was reported as 75.9% and 77%, respectively, in the ACOSOG Z1071 study and the studies conducted by Caudle et al. (15, 16). In the SenTa study, which is a multicentric German study, the SLN and CLN were found to be the same in 64.8% of the patients (27). In our study, the rate of SLN and CLN similarity was found to be 56.62% (*n* = 47), which is lower than the literature data (Figure 4). Although this is not a sufficient explanation for its clinical significance, we think it may be related to the high FNR in the SLN.

In relation to the metastasis detection rates among similar LNs, the concordance rate between SLN and CLN was 68.1% and this result was found to be statistically significant (*p* < 0.007). 16 more metastatic LNs were found in CLN than in SLN (66.3% compared to 47%, respectively) (Table 3). In a meta-analysis, the compliance rate of CLNs and SLNs was found to be between 35.7% and 87.5%, and groups with higher compliance rates were found to had superior IR and lower FNR (28).

In 12 cases double clips were placed, false negativity was detected in only one SLN, and no false negativity was detected in CLN and TAD. Although, the small sample size here creates a limitation, there is a lack of studies with larger volumes in literature. In a study conducted with fourteen patients, the FNR, which was found to be 7.1% in a single clipped lymph node, was found to be 0% when a second clipped node was added (29).

## Conclusion

Accurate axillary staging after NAC is an important issue in terms of oncological outcomes, and TAD, a technique developed for this purpose, was seen to provide safe oncological results with its feasibility and lower FNR according to the the results of our study.

## Data Availability

'The original contributions presented in the study are included in the article/Supplementary Material, further inquiries can be directed to the corresponding author.
